# Determinants of protein function revealed by combinatorial entropy optimization

**DOI:** 10.1186/gb-2007-8-11-r232

**Published:** 2007-11-01

**Authors:** Boris Reva, Yevgeniy Antipin, Chris Sander

**Affiliations:** 1 Computational Biology Center, Memorial Sloan-Kettering Cancer Center, 1275 York Avenue, New York, NY 10065, USA

## Abstract

We use a new algorithm (combinatorial entropy optimization [CEO]) to identify specificity residues and functional subfamilies in sets of proteins related by evolution. Specificity residues are conserved within a subfamily but differ between subfamilies, and they typically encode functional diversity. We obtain good agreement between predicted specificity residues and experimentally known functional residues in protein interfaces. Such predicted functional determinants are useful for interpreting the functional consequences of mutations in natural evolution and disease.

## Abstract 2

A new algorithm is presented allows protein specificity residues to be assigned from multiple sequence alignments alone. This information can be used, amongst other things, to infer protein functions.

## Background

The diversity of biologic phenomena arises from the complexity and specificity of biomolecular interactions. Nucleic acid and protein polymers encode and express biologic information through the specific sequence of polymer units (residues). The sequences and corresponding molecular structures are under selective constraints in evolution. At specific sequence position, changes in sequence alter intermolecular communication and affect the phenotype and can lead to disease [1-6]. Detailed understanding (quantitative and predictive description) of how such molecular changes affect cellular and organismic function lies at the heart of molecular and systems biology. Our ability to predict the biologic and medical consequences of human genetic variation and to design therapeutic interventions can benefit hugely from such detailed understanding. We are therefore motivated to develop further our ability to identify functionally specific residues in protein molecules.

Identifying interaction sites on protein molecules is difficult, both experimentally and theoretically. Most proteins have complicated three-dimensional shapes with interaction sites that are composed of contributions from nonsequential residues. Even with the three-dimensional structure known, however, the sites of functionally important interactions may not be obvious. Mutational experiments to probe the contributions of individual residues to such interactions are expensive. Computational methods to simulate the interactions of biologic macromolecules in molecular detail do not yet have adequate power and accuracy. Fortunately, biologic evolution has recorded rich and highly specific information in genetic sequences. For proteins, this provides the opportunity to analyze conservation patterns in amino acid sequences and extract valuable information about specific protein-partner interactions. In particular, residues in protein active sites and protein binding sites are under sufficiently strong selective pressure to allow their identification from an analysis of protein family alignments.

In a sufficiently diverse family, globally conserved residues (residues conserved in most or all family members) are easily identified and are likely to be conserved as a result of strong selective constraints. A number of research groups have developed sophisticated methods to identify additional key residues that are involved in protein structure and function, especially residues that are strongly conserved within each subfamily but differ between subfamilies [7-18]. If subfamily specific conservation patterns were perfect, then these methods would probably yield identical lists of functional residues. However, real conservation patterns can be considerably more complicated for a variety of reasons, for instance because of superimposition of multiple evolutionary constraints involving several interactions partners. In addition, current sequence collections are incomplete, for example with respect to species representation, and particular protein families are often not evenly sampled. Finally, results depend on the level of subfamily granularity (the number of subfamilies defined in a given protein family). Consequently, the extraction of biologically relevant conservation signals from multiple sequence alignments remains a challenging problem.

We present a new algorithm with which to solve the combinatorial complex problem of identifying specificity residues and, simultaneously, the corresponding optimal division into subfamilies. In our approach, called combinatorial entropy optimization (CEO), we optimize a conservation contrast function over different assignments (clusterings) of proteins to subfamilies. Hierarchical clustering [19] is used to explore the space of alternative clusterings over a diverse set of clustering trajectories to reach an optimum. Given an optimal clustering, individual residue positions (columns) vary considerably in the value of the combinatorial entropy. The distribution of column entropy values is a z-shaped curve and, reassuringly, is drastically different from the corresponding distribution for randomized alignments. Different entropy values are interpreted to reflect different residue-specific functional constraints, and residues with lowest entropy values are predicted to be functional.

We validate the method by comparing sets of predicted specificity residues with sets of experimentally known functional residues, such as interaction residues observed in three-dimensional macromolecular complexes, and we obtain good agreement between prediction and observation. Interestingly, the predictive power of the method goes beyond protein-protein interactions and is applicable to any functional constraint that conserves specific residue types in particular positions across all members of a protein subfamily.

**Figure 1:**
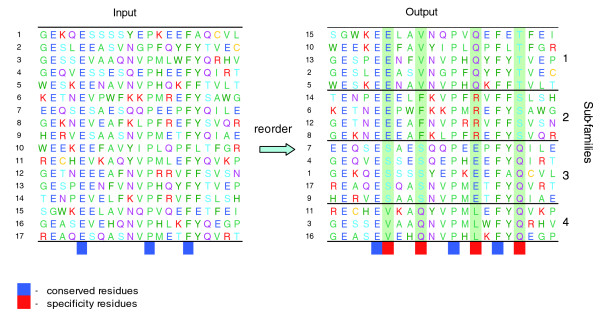
**Simple example illustrating the essence of the algorithm**. The input is a multiple sequence alignment (a protein family) in which residue conservation patterns are not obvious, except for highly conserved residues (dark blue blocks). More subtle but functionally important conservation patterns become evident after reordering the sequences and grouping them into subfamilies (output). In our algorithm, it is precisely the conservation pattern of the specificity residues (red blocks) that determines the grouping. For example, the third specificity residue is conserved as Q in the first subfamily, as R in the second, as E in the third, and as L in the fourth. An optimal subfamily arrangement of sequences has a minimal value of a sum of combinatorial entropy differences (for details, see Materials and methods).

The implementation of the method [20] takes a multiple sequence alignment as input and returns subfamilies and a set of specificity residues (Figure 1). The computed subfamilies may be used, for example, to assign a likely function to new protein sequences or to choose maximally informative targets for structural genomics projects. The computed specificity residues may be used to design highly specific mutation experiments that test function with minimal side effects; to build sharper and more informative evolutionary trees that more accurately reflect functional relatedness; to predict interactions with proteins; and to estimate the functional consequences of genetic variation.

## Results

### Parameter choice and robustness of results

The clustering algorithm partitions the sequences of a protein family into subfamilies and simultaneously selects a set of characteristic residues. The value of the contrast function, which is optimized; the number of subfamilies; and the set of the characteristic residues, which constitute the resulting optimal configuration, depend on the value of the parameter *A *(see Materials and methods, below [Equation 7]). We tested the robustness of the results with respect to parameter changes. To explore the choice of *A*, we conducted tests in a number of protein families with *A *ranging from 0.0 to 1.0, in 0.001 increments. Ideally, the selected set of characteristic residues varies slowly with *A *in a region of suboptimal *A*. The tests determined that *A *= 0.6 to 0.9 as the optimal range, and we tested all local minima of Δ*S*_0_(*A*) in this range. We tested the robustness of the results for many protein families, with representative results for two protein families in Additional data file 1. We conclude that the assignment of sequences to subfamilies is reasonably consistent with prior biologic knowledge (which in itself is incomplete and not formally defined) and that the selection of characteristic residues is reasonably stable in the range *A *= 0.6 to 0.9. For example, for protein kinases, of the top 30 characteristic residues at the overall minimum (*A *= 0.68), ranked by the column-specific difference entropies, 26 are in the top 30 at the second best local minimum (*A *= 0.72); alternatively, for ras-like small GTPases, of the top 20 residues at *A *= 0.833, 19 are in the top 20 at *A *= 0.85.

As a practical consequence of these tests, for a given protein family alignment the current software implementation of the algorithm scans the values *A *= 0.6 to 0.9 in increments of 0.025 and reports results for the value of *A *for which Δ*S*_0_(*A*) is minimum. For typical protein families this procedure yields results that resonate well with the biologic intuition of protein family experts (the reported protein subfamilies are not too fine grained nor trivially unified), and the selection of characteristic residues is a good starting point for detailed analysis and design of mutational experiments. After an initial scan, users can of course select any range of granularity parameter *A *as input and obtain more fine grained or more unified families as output.

### Validation: subfamilies and key residues of ras-like GTPases

To illustrate typical results of the CEO algorithm applied to families of amino acid sequences, we chose the small GTPases, a large and functionally diverse protein domain family with members, probably, in all eukaryotes. These GTPases are molecular switches, timed by their rate of GTP hydrolysis, which is regulated by a number of interaction partners [21]. GTPase activating proteins accelerate the GTPase by several orders of magnitude; guanine nucleotide exchange factors catalyze the binding of nucleotide after dissociation; and guanine nucleotide dissociation inhibitors stabilize the prenylated form of the GTPase in the cytoplasm and slow down dissociation of nucleotide. The switch is read out in its active form by interaction with downstream effectors, such as raf kinase for ras ad rho kinase for rho.

#### Small GTPases as testing ground

These multiple functional interactions provide an ideal testing ground for specificity analysis. A plausible evolutionary scenario involves repeated genomic duplication of an evolutionary ancestor and subsequent selection of variants, following mutation, in which the new family members have taken on a specific function. For the more than 100 distinct small GTPases in, for instance, mammalian genomes, many functions are known but our knowledge is far from complete. It is therefore interesting to analyze in which way our specificity analysis agrees with known divisions into functional protein subfamilies and to make explicit predictions pointing to candidate residues for mutational functional experiments.

#### Results for ras-like G-domains

**Figure 2:**
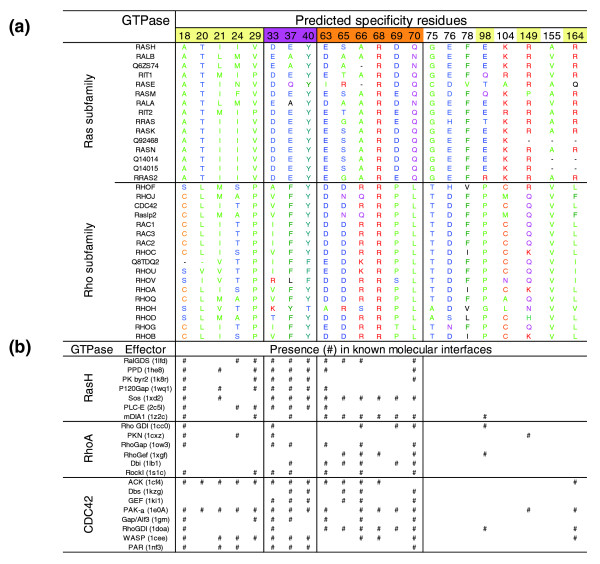
**Typical results and predictive power of the CEO method illustrated in the family of small GTPases (G-domains)**. The analysis used 126 distinct human sequences of the Ras superfamily of GTPase domains obtained after removing redundant identical copies and gappy (>30% gaps relative to rasH) sequences from the 284 protein domain sequences in the PFAM Protein Family Database (version 20), which includes ras, rab, and rho subfamilies. **(a) **Alignments of 22 specificity residues (numbered as in RasH) in the two largest ras and rho subfamilies; these residues (out of a total of about 190) carry most of the information for the distinction between functional subfamilies; note the conservation of residue type within each subfamily and nonconservation between subfamilies. **(b) **Presence of the computed specificity residues in known molecular interfaces (marked '#') of three GTPases (RasH, RhoA, and CDC42). Seventeen of the 22 specificity residues are in these interfaces (yellow numbers). Nine of the specificity residues are in the functionally important switch I (magenta numbers) and switch II (orange numbers) regions, which are involved in sensing and/or communicating the differences between the GTP and GDP states. CEO, combinatorial entropy optimization.

Our analysis of 126 unique human sequences in the Protein Families (PFAM) Ras family defines 18 subfamilies, with from 2 to 15 proteins per subfamily and 22 specificity residues that optimally discriminate between these subfamilies (Figure 2). Remarkably, a relatively small number of residues (22 out of about 200) capture the essence of subfamily discrimination, presumably as a result of functional fine tuning of interaction sites in evolution. For example (Figure 2), the following residues are characteristic for the ras/rho discrimination (amino acid numbers as in ras) D33A, E37F, S65D, A66R, D69P, and Q70L.

#### Agreement with known functional subfamilies

Because the analysis only used amino acid sequences and did not use any functional information, the concentration of similar functional names and annotations in the computed subfamilies immediately indicates successful functional classification (Additional data file 2). For example, all Ras and Rho proteins (as far as names have been assigned in the literature) are in distinct subfamilies. Finer levels of classification also appear to agree with known functional classifications; for example, Rab5A, Rab5B, and Rab5C are in a subfamiliy distinct from that of Rab6A, Rab6B, and Rab6C. As a result of systematic focus on specificity conservation patterns in our method, the implied functional distinctions between subfamilies constitute predictions when the protein class is known but functional details are not yet known.

#### Agreement with known functional residues

**Figure 3:**
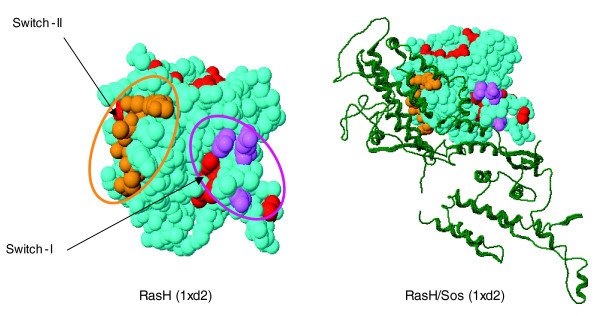
**The predicted specificity residues of the human Ras family map to known functional sites in 3D**. The specificity residues (marked '#' in Figure 2), such as the switch I and II regions, are separated along the sequence, but end up in functional positions near the active site, poised to modulate the interaction with protein partners such as the guanine nucleotide exchange factor Sos (colors as in fig. 2). Because the computation of specificity residues uses no information about known three-dimensional structures, molecular complexes, or interactions, the agreement between the computed specificity residues and their location in the experimentally observed interfaces illustrates the predictive power of the method.

Many of the 22 specificity residues in the ras family of GTPases map to well known interaction sites, triggers, and readout points of conformational change, such as the switch I region (residues 33 to 40, rasH numbering), the switch II region (residues 63 to 70), plus six additional residues (see '#'s in residue columns in Figure 2b and the corresponding placement in three dimensions in Figure 3). For some of these residues, mutation experiments have beautifully illustrated their functional importance [2,21-25], and specificity-switch experiments reveal the involvement of a few residues in favoring or rejecting a particular protein-protein interaction [26].

#### Prediction of as yet uncharacterized functional residues

Given the excellent agreement of the set of specificity residues derived from sequence family information with sets of functional residues reported as the result of detailed experiments, we are encouraged to identify potential functional residues in prediction mode. The simple hypothesis, following detailed analysis, is that all computed specificity residues have a functional implication, defined either as an observed phenotypic consequence upon changing the amino acid type or as direct observation of specific interactions (above nonspecific background) with other biologic molecules. Although such detailed predictions may be the subject of a subsequent analysis, we propose here that the following residues in the ras-type GTPases that are not in the 'switch' regions and have not been observed in protein-protein contacts in three-dimensional structures are particularly interesting (Figure 2): G75, E76, F78, K104, and A155. We propose mutational experiments for these residues within the context of carefully chosen available functional assays.

### Validation: prediction of binding sites

Various functional constraints can give rise to patterns of specificity residues, including macromolecular interfaces. To assess the predictive utility of the method for the prediction of interactions, we compared the overlap between the set of predicted specificity residues with known binding sites in several protein complexes. Although evolutionary constraints on specificity residues can be the result of any kind of functional interaction, residues in protein-protein interactions and protein-nucleic acid (NA) interactions are particularly well defined in three-dimensional structures of macromolecular complexes. A strong overlap of predicted specificity residues with binding sites would indicate that the method correctly identifies functional constraints on binding site residues. If that is the case, then one would expect a reasonable fraction of specificity residues to be binding site residues. We therefore assess the predictive potential of the implied prediction method, aware of the risk for over-prediction in cases in which other functional constraints operate outside binding sites.

#### Statistical significance and accuracy of prediction

To evaluate the overlap of predicted specificity residues (and conserved residues) with binding sites, we analyzed known three-dimensional structures of eight protein-protein/peptide complexes and five protein-NA complexes containing 19 unique proteins or protein domains belonging to 15 different so-called superfamilies from the Structural Classification of Proteins database [27]. To compute statistical significance, we compared the actual number of specificity residues in the binding site with that from a random distribution on the protein surface (see Materials and methods, below [Equation 12]). For this calculation binding site residues (interface residues) are defined as having at least one heavy (nonhydrogen) atom at a distance of 4.5 Å or less to one of the heavy atoms of the protein or NA binding partner. So what fraction of specificity residues are in protein interfaces? For example in 21 of the proteins presented in Table 1, 48% of the specificity residues are in the interfaces (and 36% of the conserved residues), with a much lower random expectation of 9% (5%); together, the specificity and conserved residues constitute about 36% of the binding interfaces (29% and 8%). The overlap is especially pronounced for protein-NA interfaces; in five protein-NA complexes 67% of the specificity residues and 35% of the conserved residues are in binding interfaces. Overall, the observed overlap is statistically significant relative to random at *P *< 0.1 in 19 out of 21 complexes (at level *P *< 0.05 in 14 complexes). In practice, interpreting specificity residues as predicted binding site residues would yield accurate predictions in about half of the cases, which is a reasonable level for planning mutational experiments. The remaining cases do not necessarily represent false-positive predictions, because other types of functional constraints, such as internal support of interaction sites or requirements of overall protein stability and correct folding, may also give rise to subfamily-specific conservation patterns. We now present specific examples of the distribution of specificity residues within the context of three-dimensional structure complexes.

**Table 1:** Statistical significance of the presence of predicted specificity residues in known interfaces of protein-protein and protein-DNA/RNA complexes

PDB^a^	Protein name^b^	Superfamily^c^	Alignment^d^	S^e^	C^e^	Ligand^f^	I^g^	S&I^g^	*P*_S&I_^g^	C&I^g^	*P*_C&I_^g^	(S+C)&I^g^	*P*_(S+C)&I_^g^
1wq1R^1 ^(1 to 166)	Ras	P-loop containing nucleoside triphosphate hydrolases	Superfamily (human) 156/0.90/0.90	13	7	1wq1G, GDP, Mg, AF3	42	8	**0.00434**	5	**0.0118**	13	**0.00007**
1wq1G^2 ^(718 to 1, 037)	P120Gap	GTPase activation domain, GAP	Superfamily (human) 20/0.90/0.90	36	15	1wq1R, GDP, Mg, AF3	33	11	**0.00024**	6	**0.00183**	17	**0**

1fvuA^3 ^(1 to 133)	Botrocetin α-chain	C-type lectin-like	Superfamily (swiss) 64/0.90/0.90	21	14	1fvuB	39	10	0.092	5	0.391	15	**0.035**
1fvuB^4 ^(401 to 525)	Botrocetin β-chain	C-type lectin-like	Superfamily (swiss) 136/0.90/0.90	29	8	1fvuA, Mg	39	13	0.077	3	0.507	16	0.0668
1a2kA^5 ^(10 to 121)	NTF2	NTF2-like	Pfam 87/0.90/0.90	18	2	1a2kD, GDP, Mg	16	7	**0.005**	0	1	7	**0.0085**
1a2kD^6 ^(12 to 170)	RAN	P-loop containing nucleoside triphosphate hydrolases	Superfamily (human) 170/0.90/0.90	17	7	1a2kA, GDP, Mg	27	6	**0.0445**	6	**0.00009**	12	**0.00004**

1i2mB^7 ^(24 to 417)	RCC1	RCC1/BLIP-II	Superfamily (nrd90) 77/0.90/0.90	45	23	1i2mA	37	10	**0.008**	0	1	10	0.089
1i2mA^8 ^(12 to 170)	RAN	P-loop containing nucleoside triphosphate hydrolases	Superfamily (human) 170/0.90/0.90	17	7	1i2mB	42	6	0.096	1	0.8	7	0.18

1rrpB^9 ^(17 to 150)	NUP358	PH domain-like	Superfamily (nrd90+swiss) 59/0.90/0.90	31	3	1rrpA	51	16	0.075	2	0.323	18	**0.032**
1rrpA^10 ^(12 to 170)	RAN	P-loop containing nucleoside triphosphate hydrolases	Superfamily (human) 170/0.90/0.90	17	7	1rrpB, GNP, Mg	53	3	0.964	6	**0.0058**	9	0.4
1blxB^11 ^(41 to 72)	P19INK4D	Ankyrin repeat	PFAM (human) 1043/0.95/0.95	7	3	1blxA	11	7	**0**	0	1	7	**0**
1blxB^11 ^(73 to 105)	P19INK4D	Ankyrin repeat	PFAM (human) 1043/0.95/0.95	7	3	1blxA	7	5	**0**	0	1	5	**0**
1blxB^11 ^(106 to 137)	P19INK4D	Ankyrin repeat	PFAM (human) 1043/0.95/0.95	7	3	1blxA	1	1	0.21	0	1	1	0.3
1blxA^12 ^(5 to 309)	CDK6	Protein kinase-like (PK-like)	Superfamily (human) 81/0.90/0.95	31	25	1blxB	24	4	0.19	0	1	4	0.19
2cciA^13 ^(4 to 286)	CDK2	Protein kinase-like (PK-like)	Protein Kinase Resource 390	20	22	1h27B1, 1h27B2, TPO	78	13	**0.0003**	11	0.0173	24	**0**
2cciB1^14 ^(181 to 307)	Cyclin A	Cyclin-like	Pfam N-cyclin 379/0.95/0.90	17	16	2cciA, 2cciF, TPO	48	12	**0.00356**	7	0.396	19	**0.0063**
2cciB2^15 ^(309 to 431)	Cyclin A	Cyclin-like	Pfam C-cyclin 238/95/90	14	3	2cciA, TPO	4	2	0.063	0	1	2	0.092
1n7tA^21 ^(14 to 98)	Erbin PDZ domain	PDZ domain	PFAM (human) 237/0.90/0.90	10	3	peptide	17	6	**0.0036**	1	0.493	7	**0.0032**
1g4dA^16 ^(13 to 81)	Repressor protein C	Putative DNA-binding domain	Superfamily (nrd90) 244/0.90/0.95	12	0	DNA	25	9	**0.0034**	0	n/a	9	**0.0034**
1e3oC^17 ^(104 to 160)	Oct-1 Pou	lambda repressor-like DNA-binding domains	Superfamily (swiss) 397/0.90/0.90	4	5	DNA	17	4	**0.00603**	3	0.151	7	**0.0018**
2up1A^18 ^(10 to 92)	Hnrnp A1, Up1	RNA-binding domain (RBD)	Superfamily (swiss) 552/0.90/1.0	16	2	DNA	21	10	**0.001**	0	1	10	**0.00166**
1ec6A^19 ^(4 to 90)	NOVA-2	Eukaryotic type KH-domain (KH-domain type I)	Superfamily (nrd90+swiss) 463/0.90/0.80	12	2	RNA	24	7	**0.019**	2	**0.074**	9	**0.0019**
1serB^20 ^(501 to 610)	Seryl tRNA synthetase	tRNA-binding arm	Superfamily (swiss) 96/0.90/0.90	18	8	tRNA	19	7	**0.022**	2	0.412	9	**0.0106**

^a^Protein Data Bank (PDB) four character code followed by the chain identifier. ^b^Name of the protein chain in the title of PDB file. ^c^Name of the corresponding Structural Classification of Proteins (SCOP) Superfamily. ^d^Source of the alignment (Superfamily or Protein Families [PFAM]); actual number of homologous sequences used in calculations, and the fractional values of the selection filters used to clean the alignments: sequence identity and gap. ^e^S and C represent the number of specificity and conserved residues, respectively. ^f^PDB identifiers of the molecular fragments and co-factors (excluding water) interacting with the corresponding protein. ^g^I, S&I, C&I, (S+C)&I stand, respectively, for the total number of interface residues (selected under ≤4.5 Å atom-atom distance threshold between ligands and the protein), the number of specificity residues in the interface, the number of conserved residues in the interface, and the number of specificity and conserved residues in the interface. *P*_S&I_, *P*_C&I_, and *P*_(S+C)&I _are the corresponding probabilities of obtaining these numbers by chance. Low values of the probabilities indicate good agreement between prediction and observation. Significant P values (< 0.05) are in bold.

### Example: interactions of cell cycle kinases

Specificity residues computed from family alignments reflect functional constraints. The distribution of specificity residues is particularly interesting for proteins engaged in multiple interactions. An example is the cell cycle kinase cyclin-dependent kinase CDK2, which plays a key role in the cell cycle (phases S and G_2_) in all eukaryotes. CDK2 forms complexes with cyclins (E and A) and specifically phosphorylates numerous substrates, such as retinoblastoma protein (pRb), retinoblastoma-like protein 1 (p107), cell division control protein CDC6, cyclin-dependent kinase inhibitor p27, tumor suppressor p53, and transcription factor E2F1. Currently, 72 proteins are reported in the Human Protein Reference Database as interacting with CDK2. CDK2 is tightly regulated; it requires specific activating phosphorylation at position Thr160 by a CDK-activating enzymatic complex (CAK); it can be inhibited by the Ink4 and Cip1/Kip1 families of cell cycle inhibitors or by phoshorylation in the glycine-rich loop by the Wee1 or Myt1 kinase. To derive specificity residues in CDK2, we used 390 sequences of protein kinases related to CDK2. We also derived specificity residues for cyclin A (379 sequences for domain N and 238 sequences for domain C).

**Figure 4:**
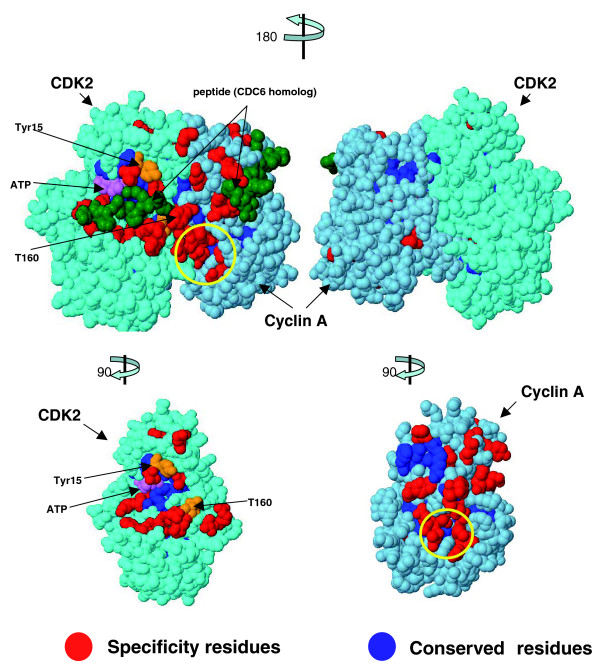
**The specificity residues in the complex of cell division protein kinase CDK2 and cyclin A**. These predicted functional residues (red and blue) are predominantly on the front (left) rather than the back (right) of the functional complex and reflect a remarkable asymmetry indicative of protein-protein interactions on the front face. We propose a novel hypothetical functional cavity on the surface of the complex (yellow circle). Other colors: green, bound peptide; orange, phosphorylation sites Y15 and T160; and pink, ATP. Coordinates from data set 2cci of the Protein Data Bank.

The distribution of specificity residues mapped to the three-dimensional structure of the CDK2-cyclin A complex is strikingly non-uniform; almost all of them are located on the 'front' face of the complex and almost none on the 'back' side (Figure 4). In addition, there are about ten specificity residues in the interface of the CDK2-cyclin A complex. This front-back asymmetry is suggestive of the assembly of a higher order complex at the front face of the CDK2-cyclin A heterodimer. On this face, specificity residues of CDK2 are in or near the following known interaction sites: phosphorylation sites T160, T14, and Y15; cyclin binding interface; and peptide substrate binding site. Some of the predicted specificity residues of cyclin A (Q228, N229, N312, Q313, T316, E330, and M334) are located in one cavity on the heterodimer surface and form a continuous molecular interface with specificity residues of CDK2 (T158 and R157). This specificity surface may reflect a previously uncharacterized binding site and may be a potential novel target site for small molecule inhibitors of CDK2-cyclin A function.

**Figure 5:**
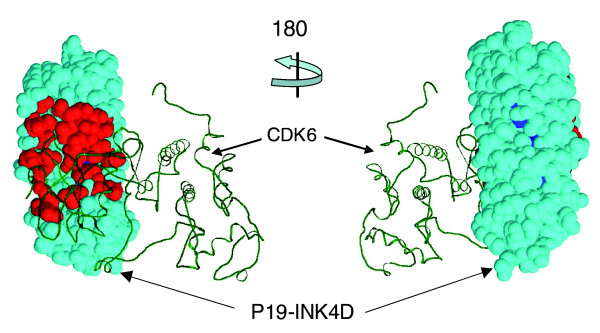
**The specificity residues of the ankyrin repeat family**. The specificity residues (red) of p19-INK4D (*CDN2D*) are concentrated on one molecular face in the three-dimensional structure (Protein Data Bank: 1blx; complex with cyclin-dependent kinase [CDK6]). As predicted, many of these residues are in the binding site. Colors as in Figure 4. The specificity residues for p19 were calculated from the PFAM alignment of ankyrin repeats and then mapped onto each of the three ankyrin repeats (residues 41 to 72, 73 to 105, and 106 to 137) of P19; the cyan structure contains all three repeats.

A related example, involving an inhibitor (p19-INK4d, gene *CDN2D*) of the cell cycle kinase CDK6, illustrates the potential power of specificity residue analysis in predicting binding site residues. The 21 specificity residues for p19-INK4d, predicted from our analysis of the alignment of 1048 human ankyrin repeats, map primarily to one patch on the surface of the molecule (Figure 5). The experimentally observed binding site, as defined by the three-dimensional structure complex of p19-INK4D with CDK6 (4.5 Å atomic proximity), overlaps with two-thirds of the residues in that patch, so the interpretation of specificity residues as predicted binding site residues would have been more than 60% accurate in this case (see Table 1 for general accuracy statistics for this prediction mode).

## Discussion

### Algorithmic innovation

The CEO algorithm is motivated by the observation that functional constraints in many cases give rise to a position-specific signature of amino acid residue types in protein sequences. Given a protein family alignment, the algorithm developed and tested here solves the challenging computational problem of detecting functional protein subfamilies and, at the same time, identifying a functional residue signature. This signature is a set of key residues (sequence positions) that vary characteristically between subfamilies but are conserved within each subfamily. The computational procedure ranks the key residues by their contribution to the optimal value of the contrast function, defined in terms of combinatorial entropy. One can use this residue ranking to prioritize further analysis and design experiments. The method also provides a signal-to-background criterion that is used to automatically classify all residues into three broad classes: specificity residues, conserved residues, and 'neutral' residues.

### Alternative solution to a complicated problem

As far as we know, the first algorithmic approaches to the problem of identification of specificity residues appeared in the mid-1990's, from the groups of Sander [7] and Cohen [8]. (See Background, above, for references to additional methods.) The current approach is sufficiently different from previous approaches to offer an alternative solution to this complicated problem. We cannot, however, claim superior performance relative to other approaches, because no 'gold standard' of experimentally determined specificity residues exists against which to validate different methods. In practice, we see a number of advantages relative to our own first approach, which was based on multivariate correspondence analysis, especially the automated definition of the resulting set of specificity residues and corresponding protein subfamilies, with granularity of subfamily division depending on a single adjustable parameter.

### Method refinement and advanced use

The algorithm performs well in practice and has been tested in many protein families in consultation with domain experts. In the future, one interesting refinement of the algorithm would be a strict distinction between paralogous (same species) and orthologous (different species) variation, provided that enough sequences are available. We are also interested in applying the method to signal enhancement in the derivation of evolutionary trees by restricting phylogenetic analysis to the subset of functionally constrained residues. Our earlier work has demonstrated the way in which evolutionary trees of this type appear less noisy and potentially reach further back in evolutionary time [7]. In another interesting application, joint specificity analysis across two protein families of potential interaction partners may lead to successful prediction of matched residues sets that are involved in protein-protein interactions [7,28]. The kernel of the CEO method may also be applicable to the analysis of gene expression patterns, patterns of gene copy number changes, and large-scale genotyping datasets. This may lead to the discovery of novel subtypes of tissues and samples, and to the derivation of characteristic genetic and molecular patterns corresponding to different developmental and disease phenotypes (Reva B, Antipin Y, Sander C, unpublished).

## Conclusion

Our results and examples demonstrate that the method can be used to identify functionally important residues from sequence information alone, without the use of three-dimensional structure or experimental functional annotation. Multiple applications are possible. The ability to locate functional determinants will be useful for the identification of residues in active sites that determine binding specificity; for the prediction of binding sites of protein complexes with other proteins, NAs, or other biomolecules; for assessing the biologic or medical significance of nonsynonymous single nucleotide polymorphisms; and for planning sharply focused mutation experiments to explore protein function. A particularly valuable application may be the design of therapeutic compounds that are highly specific to one (or a select few) of a series of paralogous proteins.

The method is publicly accessible via a web server [20] hosted in the Computational Biology Center of Memorial Sloan Kettering Cancer Center.

## Materials and methods

### Definition of the algorithmic problem

On the intuitive level, the algorithmic problem is as follows. First, divide a given multiple sequence alignment into subfamilies (also called sequence clusters) such that each subfamily has a characteristic conservation signature at a number of sequence positions. Then, optimize the information in the subfamily division to achieve a reasonable compromise between the number of proteins in a subfamily and the number of characteristic residues positions used to distinguish the subfamilies from each other (the larger the number of proteins per subfamily, the smaller the number of characteristic residue positions, and *vice versa*; the two extremes of 'one sequence per subfamily' and 'all sequences in a single subfamily' are uninformative).

To solve this problem, one must introduce a measure to compare different distributions of sequences into subfamilies. The simplest measure is additive for the columns in the alignment. This means that the distribution of residues in alignment columns within a subfamily is treated independently (all possible permutations of residues in a column within a subfamily are equivalent). The total number of permutations in a column *i *of a subfamily *k *is given by a simple combinatorial formula [29]:

$$Z_{i,k}=\frac{N_{k}!}{\prod_{\alpha =1,...,21}N_{\alpha ,i,k}!}$$

Here *N*_*k *_is the number of sequences in subfamily *k*; N_*α*,*i*,*k *_is the number of residues of the type *α *in column *i *of subfamily *k*. (Gaps are taken into account as a separate residue type; *α *= 21 corresponds to a gap.) The numerator is the total number of permutations of *N*_*k *_symbols and the product in the denominator divides out the number of indistinguishable permutations for each residue type α.

We then use the statistical or combinatorial entropy [29]:

$$S=\sum_{i}S_{i}$$

Where

$$S_{i}=\sum_{k}\ln Z_{i,k}$$

is an additive measure (both in terms of alignment columns and subfamilies) for comparing different distributions of residues. The statistical entropy depends on subfamily size. The entropy of the union of two subfamilies is always greater than or equal to the sum of entropies of the individual subfamilies. The entropy is equal to zero when all sequences are separated into subfamilies of a single sequence each (maximal fragmentation); the entropy is maximal when all sequences are united in one family (maximal unification). The dependence of the statistical entropy on subfamily sizes allows one to formulate an optimization problem, namely find the distribution of sequences into subfamilies that is maximally different from a random distribution of sequences. Subfamilies of sequences with many conserved residue patterns (which change across subfamilies) will contribute the most to the optimal solution.

We define specificity residues (also called characteristic or key residues) as residues that are conserved in a subfamily but differ between subfamilies. Thus, one is challenged to determine simultaneously the best division of the set of sequences into subfamilies and the subset of residues that best discriminates between these subfamilies. 'Best' is defined in terms of a contrast function that aims to measure the degree to which the specificity residues are distinctly different in each subfamily. The value of the contrast function is minimal for the best solution, with the result reported as a set of specificity residues and corresponding sequence subfamilies. The sections below describe the contrast function, the meaning of 'best', the optimization algorithm, and a criterion for selecting the top-ranked specificity residues.

### Definition of the contrast function in terms of combinatorial entropy

Suppose a multiple alignment is divided into subfamilies or clusters of sequences. For each column *i *(*i *= 1, ..., *L*) of the alignment, one can compute the combinatorial entropy *S*_*i*_, as defined by Equation 3 (above). At one extreme, the column-specific *S*_*i *_is zero if residues of one type populate this column in each of the clusters, no matter whether this residue type is the same in all clusters or differs between clusters (for example, see the specificity residue columns in Figure 1). So *S*_*i *_= 0 for completely conserved residues or perfect specificity residues in column *i*. At the other extreme, for uniformly distributed residues, *S*_*i *_has a maximal value given by the background entropy ${\tilde{S}}_{i}$

$${\tilde{S}}_{i}=\sum_{k}\ln{\tilde{Z}}_{i,k}=\sum_{k}\frac{N_{k}!}{\prod_{\alpha =1,...,21}{\tilde{N}}_{\alpha ,i,k}!}$$

Where ${\tilde{N}}_{\alpha ,i,k}$ is the expected number of the residues of a type *α *in the column *i *of the subfamily *k*, provided that all the residues in the column are uniformly mixed (across column boundaries), namely where

$${\tilde{N}}_{\alpha ,i,k}=N_{k}N_{\alpha ,i}/N$$

and *N*_*α*,*i *_is the number of residues of type α in column *i *and *N *is the total number of sequences (lines) in alignment. (Because ${\tilde{N}}_{\alpha ,i,k}$ can be noninteger numbers, ${\tilde{N}}_{\alpha ,i,k}$! is computed using the relation *X*! = Γ(*X *+ 1) [30].)

As the numerical measure of order over disorder, the entropy difference Δ*S*_*i *_= *S*i - ${\tilde{S}}_{i}$ between the observed and uniformly mixed distribution, summed over all *L *columns of the alignment:

$$\Delta S_{0}=\sum_{i=1,...,L}\Delta S_{i}$$

is the contrast function to be minimized in the process of finding the best decomposition into subfamilies. (Because Δ*S*_0 _is a negative number, this means that the absolute value of Δ*S*_0 _is maximized.)

### The optimization algorithm

A straightforward solution to the optimization problem would be to enumerate all possible partitionings of the set of sequences into subfamilies, calculate the combinatorial entropy difference (the contrast function) as in Equation 6, and then choose the partitioning with the lowest value of Δ*S*_0_. The only problem with this approach is that the number of partitionings of *N *sequences into *K *clusters is astronomically large for all but very small values of *N *and *K*. One therefore needs an effective strategy for exploring a reasonable subset of partitonings with the aim of finding one with a value of the contrast function close to the global optimum. Often such complex value landscapes are explored using stochastic algorithms, which can be used in future implementations. In this report we use a simple deterministic hierarchical clustering method [19] with each clustering step guided by evaluation of a guide function (Equation 7) for all alternative choices in that step.

Starting from *N *clusters, each containing one sequence, in each clustering step all pairs of clusters are considered as merger candidates. The pair of clusters with the lowest value of the guide function is merged into one cluster. The merger steps are repeated until all sequences are in one cluster. At this stage the result is a complete trajectory of merger steps, which can be represented as a tree (not shown) and the task is to choose the best partioning (tree level). The best partioning is defined as the one with the minimal value of Δ*S*_0_, or the maximal absolute value of the combinatiorial entropy difference between the actual and uniformly mixed ('random') distribution of residue types (Equation 6). The complexity of the hierarchical clustering algorithm is of *O*(N**2 ln N), where *N *is the number of sequences in the multiple alignment [31].

To explore different partitionings of sequences into subfamilies, the guide function includes a penalty term [32]. The penalty term affects the clustering trajectory by favoring mergers that result in smaller clusters over those that result in larger clusters. To explore a larger space of alternative partionings, we perform hierarchical clustering for different relative weights of the penalty term.

The guide function used to evaluate a particular clustering step (potential merger of clusters *k *and *m*) is defined as follows:

$$\Delta Q_{k,m}=A\Delta S_{k,m}+(1-A)\Delta S_{k,m}^{'}$$

The first term, Δ*S*_*k*,*m*_, is the entropy difference computed for the new cluster resulting from the merger of clusters *k *and *m*:

$$\Delta S_{k,m}=\frac{1}{L}\sum_{i=1,...,L}\ln\prod_{\alpha =1,...,21}\frac{({\tilde{N}}_{\alpha ,i,k}+{\tilde{N}}_{\alpha ,i,m})!}{(N_{\alpha ,i,k}+N_{\alpha ,i,m})!}$$

averaged over all *L *columns of the alignment.

The second term, $\Delta S_{k,m}^{'}$, the penalty term, makes reference to the combinatorial entropy of an ideal system of the same size:

$$\Delta S_{k,m}^{'}=\ln(N_{k}+N_{m})!$$

Where *N*_*k *_and *N*_*m *_are the number of sequences in the corresponding clusters *k *and *m*.

Δ*S'*_*k*,*m *_is the maximal possible value of the combinatorial entropy (per column) after merging clusters of size *N*_*k *_and *N*_*m*_. This second term simply captures the mere size contribution to the entropy and counteracts the tendency toward trajectories with early emergence of dominant large clusters. This tendency is due to the fact that the entropy of a larger system is always greater than the sum of the entropy values of its subsystems. Whatever the trajectories explored and whatever the devices used to guide the exploration of trajectory space, the evaluation of best partitioning is exclusively based on the combinatorial entropy difference of Equation 6.

**Figure 6:**
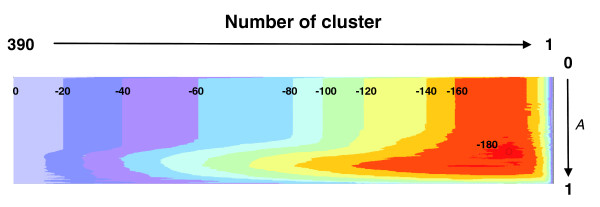
**Value landscape of the contrast function for a large protein family illustrating the optimization process**. The algorithm searches for the minimal value of the contrast function (a combinatorial entropy difference [Equation 6]) by systematic exploration of different clusterings (horizontal axis) and of different values of the granularity parameter *A *(vertical axis). The overall minimum (circle in red area, lower right, *A *= 0.68, value of normalized contrast function -187) determines which protein is in which subfamily and which residues contribute most to the specificity patterns across the subfamilies. Here, the value landscape (color contours, values normalized by the number of residues [283 columns] in the alignment) was computed for a multiple alignment of 390 protein kinases [36] with 0.0 <*A *< 1.0. Note that the lowest entropy value at *A *= 1 is far from the overall minimum, indicating the utility of this parameter.

Extreme values of the granularity parameter *A *in Equation 7 lead to radically different trajectories in clustering space. When *A *is approximately 1, the main contribution to the guide function of Equation 7 comes from the entropy difference due to sequence assignment to subfamilies; when *A *is approximately 0, clustering is driven by cluster size and mergers into smaller clusters are favorable. Changing the granularity parameter *A *in the guide function over a reasonable range of values and repeating hierarchical clustering explores sufficiently diverse partitionings to reach an optimum (Figure 6).

Note that although the guide function determines the details of each clustering step, the final optimum is chosen as the minimum of the combinatorial entropy difference (Equation 6) in the two-dimensional space of two variables, the clustering step *l*, and the penalty weight (1 - *A*). Typical optimal values of *A *in tests for diverse protein families range between 0.6 and 0.9.

### Evidence for selective pressure and selection of specificity residues

**Figure 7:**
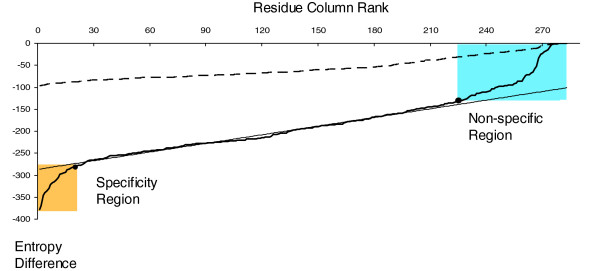
**Definition of specificity residues based on entropy values**. Combinatorial entropy difference as a function of residue position (in rank order) for the actual (solid line) and randomized (dashed line) multiple alignment of 390 protein kinase sequences [36]. Deviations from the linear fit to the entropy curve define the specificity region (yellow, about 20 residues, conserved in subfamilies but varying between subfamilies) and conserved region (blue, about 50 residues, conserved across all subfamilies). The randomized alignment, obtained by independently shuffling residues in each column of the original alignment, serves as a point of reference. The shuffling does not affect the residue content in the columns, but it washes out the subfamily distinctions. The greater the differences between the native and the randomized entropy curves, the more reliable the corresponding prediction of specificity residues. To automate visual parsing of the extreme ends of the entropy plots, we perform a simple linear fit to the central region, covering a fraction *P *= 0.5 to 0.7 (depending on the length of the alignment) of the sequence length (horizontal range). The line segment is centered at a point corresponding to the best linear fit. To identify the turning points at the extremes, we compute the root mean square deviation $\delta_{p}=\sqrt{<{\left(\Delta S_{i}-<\Delta S>\right)}^{2}>}$ from a simple line in the central region and record the points outside of the central region where the curve deviates by more than *δ*_*p *_from the extrapolated line segment. In most cases, this simple procedure is in agreement with visual identification of downturn and upturn at the extremes. A reasonable subset of specificity residues (low end of entropy difference) and conserved residues (high end) can then be read off from the horizontal axis of the entropy plot.

Selective pressure in evolution results in patterns of conservation across all subfamilies (globally conserved residues) or in particular subfamilies (specificity residues). Examples of conserved residues are active site residues in enzyme families, and examples of specificity residues are residues lining active sites configured to bind a particular substrate optimally. The combinatorial entropy difference (Equation 6) is greatest for alignment columns with specificity residues (by definition; see above), but close to zero for 'nonspecific' columns that do not discriminate between subfamilies. Such 'nonspecific' columns have globally conserved residues or diverse nonspecific residue distributions. All other residue columns have intermediate values of Δ*S*_0_. Thus, if we sort residue columns by their entropy difference Δ*S*_0 _and plot the resulting distribution (Figure 7), then we can typically identify two regions of particularly low and particularly high entropy difference Δ*S*_0_. For typical alignments, one can visually identify the characteristic extreme regions of the entropy as deviations from the linear central region.

We compared entropy plots for the original alignment with the entropy plot for a randomized alignment (for details, see Figure 7). The differences between the original and the randomized entropy plots are drastic; there are no downturn and upturn regions in the entropy plots for randomized alignments, and the absolute values of the entropy differences produced for the randomized alignments are several times smaller than those of the original alignments.

To distinguish between globally conserved columns and other 'nonspecific' columns, we compute the combinatorial entropy for each alignment column:

$$S_{i}=\ln\frac{N!}{\prod_{\alpha =1,...,21}N_{\alpha ,i}!}\approx -N\sum_{\alpha =1,...,21}\frac{N_{\alpha ,i}}{N}\ln\frac{N_{\alpha ,i}}{N}=-N\sum_{\alpha =1,...,21}f_{i,\alpha }\ln f_{i,\alpha }=N{\langle s\rangle }_{i}$$

Where

$${\langle s\rangle }_{i}=-\sum_{\alpha =1,...,21}f_{i,\alpha }\ln f_{i,\alpha }$$

is the average entropy per residue for the residue distribution in alignment column *i*; *f*_α,*i *_is the fraction of residues of type α in column *i *(*α *= 21 for gaps). We require ⟨*s*⟩_*i *_< 0.03 and *f*_21,*i *_< 0.5 for globally conserved columns; mathematical details related to Equations 10 and 11 are provided in Additional data file 3.

### Test application: prediction of contact residues and evaluation of accuracy

Specificity residues - and, of course, globally conserved residues - reflect functional constraints that operate in evolution. They are an informational fossil record, most clearly visible over large evolutionary intervals during which the background distribution may vary considerably. The constraints can be of diverse origin, but it is plausible that all constraints can be traced to the requirements of intermolecular interactions that are important for survival. Therefore, prediction of specificity residues has broad applicability for the identification of functional interactions and, as a consequence, for ranking genetic variation, for planning mutation experiments, or for the molecular design of specificity.

Here, we test one particular application of the identification of specificity residues from multiple sequence alignments: the prediction of intermolecular interfaces. We use known three-dimensional structures of protein and DNA complexes from the Protein Data Bank (PDB) as defining experimental reality against which predictions are compared. A key limitation is that there may be several such interfaces in a given protein family and that the complexes in the PDB contain only a subset of these. Nonetheless, it is instructive to see the extent to which specificity residues, interpreted as predicted interface residues, overlap with known intermolecular interfaces. A large overlap indicates good prediction accuracy, but over-prediction (false positives) is expected.

To assess whether an observed overlap between specificity residues and intermolecular interface residues is statistically significant, we estimate the expected size of overlap in a random model, in which specificity residues are scattered randomly in the protein and may or may not end up in the known interface by chance. Suppose that the total number of protein residues is *N*, the number of the known interface residues is *L*, the number of the specificity residues is *S*, and the number of the specificity residues in the interface is *A*. If the specificity residues are randomly distributed, then what is the probability of observing *A *or more of the *S *specificity residues in the interface? For reasons of permutational degeneracy, one must compute the total number of indistinguishable variants of *A *distinct residues assigned to four sets of size *K*, *M*, *J *and (*N *- *K *- *M *- *J*) residues:

$$Z(N,K,M,J)=\frac{N}{K!M!J!(N-K-M-J)!}$$

Then, the probability to observe at random *A *or more of *S *specificity residues among the *L *interface residues is given by the following ratio:

$$P(N,L,S,A)=\frac{\sum_{Q=A}^{\min(L,S)}Z(N,L-Q,S-Q,Q)}{\sum_{Q=0}^{\min(L,S)}Z(N,L-Q,S-Q,Q)}$$

Where the numerator represents the number of all possible assignments for which the sets of size *S *and *L *have *A *or more common residues; and the denominator represents the total number of all possible assignments up to complete overlap of the two sets. To correct for the *N*_*c *_globally conserved residues, which by definition are excluded from being identifies as specificity residues, we use *N *- *N*_*c *_in Equation 12 in place of *N*.

### Choice of multiple sequence alignments

The multiple sequence alignments are the only source of information used in the predictions. Predictions are best for accurate, nonredundant alignments of diverse sequences without significant gap regions. In the interface prediction tests, we used alignments from the 'Superfamily' [33] and PFAM [34] collections, as well as the Homology-Derived Secondary Structure of Proteins database [35] and curated alignments of human protein kinases [36] from the Protein Kinase Resource [37]. As needed, the original alignments were prepared for specificity analysis by trimming deletions and insertions across the whole alignment so as to preserve the continuity of the main sequence (the sequence of a given protein); removing redundant sequences (typically at the level of about 95% identical residues for large alignments) using the MView program [38,39]; and removing sequences with many gaps (for example, with more than about 10% to 20% gaps compared with the main sequence). Finally, the total number of sequences in the alignment must be large (>100).

## Abbreviations

CDK, cyclin-dependent kinase; CEO, combinatorial entropy optimization; NA, nucleic acid; PDB, Protein Data Bank; PFAM, Protein Families.

## Authors' contributions

BR and CS specified the problem and developed the algorithm. BR and YA wrote the software and performed the data analysis. All wrote the paper.

## Additional data files

The following additional data are available with the online version of this paper. Additional data file 1 is a table summarizing the results of a robustness analysis of the method, as described in the main text. Additional data file 2 is a table summarizing the results of optimal clustering of 126 GTPases of human Ras superfamily. Additional data file 3 is a tutorial section that explains the link between the common notion of probability entropy (information entropy) and the less well known formulation of combinatorial entropy.

Source code of the core method is available on request from the authors, subject to acceptance of a public domain license.

## Supplementary Material

Additional data file 1 Presented is a table reporting the results of a robustness analysis of the method, as described in the main text. Click here for file

Additional data file 2 Presented is a table reporting the results of optimal clustering of 126 GTPases of the human Ras superfamily. Click here for file

Additional data file 3 Presented is a tutorial section that explains the link between the common notion of probability entropy (information entropy) and the less well known formulation of combinatorial entropy. Click here for file
