# Supplementary material for: Determinants of protein function revealed by combinatorial entropy optimization
Source: Genome Biol. 2007 Nov 1;8(11):R232. doi: 10.1186/gb-2007-8-11-r232 (PMC2258190; doi:10.1186/gb-2007-8-11-r232)
Supplement: Additional data file 1 — Presented is a table reporting the results of a robustness analysis of the method, as described in the main text. [file gb-2007-8-11-r232-S1.pdf]

Table SI. Robustness of the top-ranked alignment columns obtained at different values of ***A***.

Alignment[a]	A	ΔS^{b}_{opt}	$<n_{cl}>^{c}$	P_{10}^{d}	P_{20}^{d}	P_{30}^{d}
Kinases	0.5	-178	10.8	8	16	26
	0.58	*-177*	*10.5*	9	16	25
	0.63	-186	11.1	8	18	26
	0.66	*-182*	*8.9*	10	17	26
	**0.68**	**-187**	**10.8**	**10**	**20**	**30**
	0.71	*-184*	*11.1*	9	19	25
	0.72	-186	13	8	17	26
	0.75	*-184*	*11.5*	8	17	26
	0.76	-185	13.4	9	18	26
	0.8	-182	13.9	9	18	25
	0.9	-169	8	7	13	23
Ras	0.6	*-255*	*13.6*	9	18	27
	0.7	-264	12.3	9	19	26
	0.77	*-259*	*10.5*	9	18	27
	**0.833**	**-267**	**8.6**	**10**	**20**	**30**
	0.84	*-266*	*8.2*	9	20	29
	0.85	-267	8.9	9	19	30
	0.9	-249	8.7	8	18	27

[a]Alignment of 390 kinase catalytic domains was taken from the kinase sequence database of Hanks and Quinn [35,36]; alignment of 515 sequences of Ras homologs were taken from HSSP database [34]; [b]Entropy difference are computed by Eq.6 and normalized by a number of columns in the alignment; [c]$<n_{cl}>$ stands for an average number of sequences in a cluster; [d]P_{10}, [d]P_{20}, [d]P_{30} are the numbers of top-ranked columns that are commonly determined for a given and for the minimal entropy clustering; the numbers of the common columns are computed for the 10, 20 and 30 top ranked positions; the optimal clustering data are highlighted.
