# Supplementary material for: Determinants of protein function revealed by combinatorial entropy optimization
Source: Genome Biol. 2007 Nov 1;8(11):R232. doi: 10.1186/gb-2007-8-11-r232 (PMC2258190; doi:10.1186/gb-2007-8-11-r232)
Supplement: Additional data file 3 — Presented is a tutorial section that explains the link between the common notion of probability entropy (information entropy) and the less well known formulation of combinatorial entropy. [file gb-2007-8-11-r232-S3.pdf]

Supplement

Entropy of the residue distribution in an alignment column and the relationship between combinatorial and statistical entropy

This tutorial section provides a link between the common notion of *probability* entropy and the less well known formulation of *combinatorial* entropy. Given a sequence alignment, in which rows represent proteins and columns represent residues, the diversity of a residue distribution in an alignment column i is typically evaluated by the *probability* entropy:

$$\langle s_i \rangle = -\sum_a f_{a,i} \ln f_{a,i} \qquad \text{(S1)}$$

where $f_{a,i} = N_{a.i} / N$ is the fraction of a column i occupied by residues type a ($N_{a.i}$ is a number of residues type a in a column i; N is the total number of sequences in alignment). In this paper, we use an alternative, but related measure of residue diversity, the *combinatorial* or *statistical* entropy. We now discuss the relation between the combinatorial and probability formulation of entropy and discuss the applicability of both entropies to the task of clustering sequence alignments.

The statistical entropy is defined as follows [1]

$$S = k_B \ln Z\,, \qquad \text{(S2)}$$

where Z is the number of microstates consistent with a given macrostate, and k_B is Boltzmann's thermodynamic constant (this constant provides units conversion and can be omitted for the sake of simplicity). Applied to sequence alignments or other character tables, a *microstate* is simply one particular distribution of residues in the alignment column of the given subfamily, while the *macrostate* is defined by the total numbers of residue types, $N_{a.i}$. All microstates have equal statistical weights. The total number of microstates in an alignment column i of a subfamily m is given by

$$Z_i^m = \frac{N^m!}{\prod_{a=1,\dots,21} N_{i,a}^m} \qquad \text{(S3)}$$

where $N_{i,a}^{m}$ is a number of residues type a ?in an alignment column *i* of a subfamily *m* (*m* is an index, not an exponent) ; $N^{m} = \sum_{a=1,..,21} N_{i,a}^{m}$ is the size of a subfamily *m*; (it is the same for all columns) the sum is taken over all 20 types of amino acid residues and gaps (α=21 refers to a gap).

The classical statistical entropy of Eq.S2 obviously depends on the size of the system; it provides a natural measure for comparing different groupings of sequences into subfamilies. We use Eqs.S2-S3 to introduce a "contrast function" – the difference between the entropy of a particular grouping of sequences into subfamilies and the entropy of non-specific or "uniform" grouping of sequences into subfamilies of the same size (see Eq.6 in the main text). The optimal value of the contrast function corresponds to optimally ordered sequences into subfamilies.

The link between the combinatorial and probability formulation becomes apparent via a simple mathematical approximation. Using Sterling's formula $N! \approx \sqrt{2pN}(N/e)^{N}$ in the logarithmic form $\ln N! \approx N(\ln N - 1)(1 + \frac{1}{N}\frac{\ln N + \ln 2p}{2\ln(N-1)}) \approx N(\ln N - 1)$, when $N >> 1$, one obtains:

$$S_{i}^{m} = \ln \frac{N^{m}!}{\prod_{a=1,...,21} N_{i,a}^{m}} = -\ln \frac{\prod_{a=1,...,21} N_{i,a}^{m}!}{N^{m}!} \approx -N^{m} \sum_{a=1,...,21} \frac{N_{i,a}^{m}}{N^{m}} \ln \frac{N_{i,a}^{m}}{N^{m}} = -N^{m} \sum_{a=1,...,21} f_{i,a}^{m} \ln f_{i,a}^{m} = N^{m} \langle s_{i}^{m} \rangle , \quad \text{(S4)}$$

where $f_{i,a}^{m} = N_{i,a}^{m} / N^{m}$ is the fraction of residues of type a in column *i* of subfamily *m:* and

$$\langle s_{i}^{m} \rangle = -\sum_{a=1,...,21} f_{i,a}^{m} \ln f_{i,a}^{m} \quad \text{(S5)}$$

is the average entropy of residue distributions in the column *i* of the subfamily *m*.

It is easy to see that $\langle s \rangle = 0$, when an alignment column is completely conserved, and $\langle s \rangle = \ln 20 \approx 3$, if all 20 types of residues are equally present in a gapless column. Therefore the average entropy $\langle s_{i}^{m} \rangle$ is often used to evaluate the diversity of a residue

distribution in an alignment because the value of $q_i^m = \exp(\langle s_i^m \rangle)$ provides an estimate of the number of "microstates" i.e. the number of different residue types per position in the alignment column. The relation $S_i^m = N^m \langle s_i^m \rangle$ (Eq.S4) is only valid if all $N_{i,a}^m$ are large. Therefore for small clusters it is essential to use the more informative equations S2-S3 rather than the approximation of Eq.S4.

Note that the average entropy $\langle s \rangle$ of Eq.S5 does not depend on the size of the system and therefore it cannot be applied to evaluate clustering of sequences into subfamilies. In addition, using the average entropy $\langle s \rangle$ instead of the statistical entropy S (Eqs.S2-3) one may obtain a nonsensical result in which the entropy of two merged systems is smaller than a sum of entropies of individual systems, i.e. $\langle s^{1+2} \rangle < \langle s^1 \rangle + \langle s^2 \rangle$.

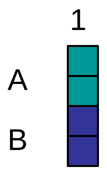

$$f_A^1 = N_A^1/(N_A^1 + N_B^1) = \frac{2}{2+2} = \frac{1}{2};$$
$$f_B^1 = N_B^1/(N_A^1 + N_B^1) = \frac{2}{2+2} = \frac{1}{2};$$
$$\langle s^1 \rangle = -(f_A^1 \ln f_A^1 + f_B^1 \ln f_B^1) = -(\frac{1}{2}\ln\frac{1}{2} + \frac{1}{2}\ln\frac{1}{2}) = \ln 2$$

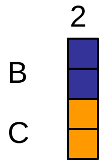

$$f_B^2 = N_B^2/(N_B^2 + N_C^2) = \frac{2}{2+2} = \frac{1}{2};$$
$$f_C^2 = N_C^2/(N_B^2 + N_C^2) = \frac{2}{2+2} = \frac{1}{2};$$
$$\langle s^2 \rangle = -(f_B^2 \ln f_B^2 + f_C^2 \ln f_C^2) = -(\frac{1}{2}\ln\frac{1}{2} + \frac{1}{2}\ln\frac{1}{2}) = \ln 2$$

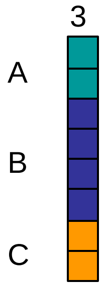

$$f_A^3 = N_A^3/(N_A^3 + N_B^3 + N_C^3) = \frac{2}{2+4+2} = \frac{1}{4};$$
$$f_B^3 = N_B^3/(N_A^3 + N_B^3 + N_C^3) = \frac{4}{2+4+2} = \frac{1}{2};$$
$$f_C^3 = N_C^3/(N_A^3 + N_B^3 + N_C^3) = \frac{2}{2+4+2} = \frac{1}{4};$$
$$\langle s^3 \rangle = -(f_A^3 \ln f_A^3 + f_B^3 \ln f_B^3 + f_C^3 \ln f_C^3) = -(\frac{1}{4}\ln\frac{1}{4} + \frac{1}{2}\ln\frac{1}{2} + \frac{1}{4}\ln\frac{1}{4}) = \frac{3}{2}\ln 2$$

Figure S1

Indeed, suppose a cluster 1 is composed of 2 residues A and 2 residues B; a cluster 2 is composed of 2 residues B and 2 residues C; the corresponding probability entropies of both clusters, $\langle s^1 \rangle$ and $\langle s^2 \rangle$ are equal to ln2. Merging these clusters (see details in Fig.S1), one obtains a cluster 3 with 2 residues A, 4 residues B and 2 residues C that

results in the probability entropy $\langle s^3 \rangle = \frac{3}{2}\ln 2 < \langle s^1 \rangle + \langle s^2 \rangle = 2\ln 2$. Hence, in this example, the entropy of the union of two systems is smaller than the sum of the entropies of the original systems, which is inconsistent with the principle (expectation) that the information content of a combined system is larger or equal to the sum of the information content of its parts [1]. We conclude that the statistical entropy rather than the average (probability) entropy should be used. Indeed, using the definition of statistical entropy in Eq.S1, one obtains: $S_1 = S_2 = \ln \frac{4!}{2!2!} = \ln 6 \approx 1.8$; $S_3 = \ln \frac{8!}{2!4!2!} = \ln 420 \approx 6$. This gives $S_3 > S_1 + S_2$, as it should be.

References

1. Landau LD and Lifshitz EM: **Statistical Physics**, 3rd Edition Part 1", Butterworth-Heinemann, Oxford, 1996.
